# Credal decision tree based novel ensemble models for spatial assessment of gully erosion and sustainable management

**DOI:** 10.1038/s41598-021-82527-3

**Published:** 2021-02-04

**Authors:** Alireza Arabameri, Nitheshnirmal Sadhasivam, Hamza Turabieh, Majdi Mafarja, Fatemeh Rezaie, Subodh Chandra Pal, M. Santosh

**Affiliations:** 1 Department of Geomorphology, Tarbiat Modares University, Jalal Ale Ahmad Highway, 9821 Tehran, Iran; 2 Faculty of Geo-Information Science and Earth Observation (ITC), University of Twente, Enschede, The Netherlands; 3 Department of Geography, Bharathidasan University, Tiruchirappalli, Tamil Nadu 620024 India; 4 Department of Information Technology, College of Computers and Information Technology, Taif University, P.O. Box11099, Taif, 21944 Saudi Arabia; 5 Department of Computer Science, Birzeit University, Birzeit, Palestine; 6 Geoscience Platform Research Division, Korea Institute of Geoscience and Mineral Resources (KIGAM), 124, Gwahak-ro Yuseong-gu, Daejeon, 34132 Republic of Korea; 7 Korea University of Science and Technology, 217 Gajeong-roYuseong-gu, Daejeon, 34113 Republic of Korea; 8 Department of Geography, The University of Burdwan, Bardhaman, West Bengal 713104 India; 9 School of Earth Sciences and Resources, China University of Geosciences Beijing, Beijing, China; 10 Department of Earth Sciences, University of Adelaide, Adelaide, South Australia Australia

**Keywords:** Environmental sciences, Hydrology, Solid Earth sciences

## Abstract

We introduce novel hybrid ensemble models in gully erosion susceptibility mapping (GESM) through a case study in the Bastam sedimentary plain of Northern Iran. Four new ensemble models including credal decision tree-bagging (CDT-BA), credal decision tree-dagging (CDT-DA), credal decision tree-rotation forest (CDT-RF), and credal decision tree-alternative decision tree (CDT-ADTree) are employed for mapping the gully erosion susceptibility (GES) with the help of 14 predictor factors and 293 gully locations. The relative significance of GECFs in modelling GES is assessed by random forest algorithm. Two cut-off-independent (area under success rate curve and area under predictor rate curve) and six cut-off-dependent metrics (accuracy, sensitivity, specificity, F-score, odd ratio and Cohen Kappa) were utilized based on both calibration as well as testing dataset. Drainage density, distance to road, rainfall and NDVI were found to be the most influencing predictor variables for GESM. The CDT-RF (AUSRC = 0.942, AUPRC = 0.945, accuracy = 0.869, specificity = 0.875, sensitivity = 0.864, RMSE = 0.488, F-score = 0.869 and Cohen’s Kappa = 0.305) was found to be the most robust model which showcased outstanding predictive accuracy in mapping GES. Our study shows that the GESM can be utilized for conserving soil resources and for controlling future gully erosion.

## Introduction

The agrarian economy is faced with the challenge of maintaining food security despite the increasing global population, and in tackling serious threats, including a decline in food productivity, climate change and lack of freshwater resources^1^. Better conservation of soil resources, which necessitates control on soil erosion, is one of the most significant aspects in improving land productivity^2^. Soil is a finite resource and plays a major role in human existence as the source of more than 99% of our nourishment^3^. Among several triggering agents for soil erosion, water plays a major role^2^. It has been assessed that soil erosion causes a yearly global GDP loss of almost $8 billion^2^. Iran is among the many countries that is worst affected by soil erosion, with an annual soil loss of about 32 tons per hectare from farmlands^3^. The most adverse type of water-triggered soil erosion that largely deteriorates the agricultural lands of Iran is gully erosion (GE)^2^.

Gullies can be temporary (ephemeral) or permanent (classical) where the latter is larger than the former^9^. In places where intense flow intersects earth bank, bank gullies can also occur. In general, gullies represent incised deep linear geomorphological features, varying in depth between 0.5 and 30 m^4^. Development of gullies mostly occur in loess soil^5^. There are two phases in gully development, one is initiation of gully which occurs in smaller timespan and the other is the stable sediment transportation phase^2^. GE is created by running water, mass-wasting and subterranean process that erodes soil particles^6^, and results in numerous onsite and offsite effects including land degradation, soil fertility loss, and accumulation of sediments, landslide, flooding and decline of water quality^5–7^. GE not only causes environmental deterioration but also immensely impacts the socio-economic aspects^8^. Previous studies have shown the main role of GE in transporting sediments from upper region of the catchments^9^. Thus, a precise evaluation of gully erosion susceptibility (GSE) is an essential requirement for planners and decision-makers in controlling the subsequent problems of GE and for a sustainable management of soil resources^3^.

Various factors including topographic, geologic, hydrologic, environmental, climatic and anthropogenic activities, instigate the process of GE^10–12^. Rahmati et al.^10^ reported that drainage density, distance to stream and land use also play a vital role in triggering GE. Zhao et al.^12^ noted that GE is mostly initiated by natural processes rather than anthropogenic activities and that the density of gullies is reliant on the intensity of vegetation cover and topographic features.

Most of the physically based models reported in earlier studies of gully erosion were not aimed at predicting the gully hotspots, but focused on quantifying the erosion rates^11^. For predicting the evolution of gullies, dynamic and static models have been utilized previously based on the development phase of the gully^2^. However, both these models require different erosion factors which are hard to quantify for a large area. Thus, for the gully erosion susceptibility mapping (GESM), researchers utilized various models such as knowledge based, statistical and machine learning algorithms (MLAs) coupled with geographical information system (GIS) and remote sensing (RS)^13^. The knowledge-based models include multi-criteria decision-making models (MCDM) that involve the decision made by experts to prepare the GESM. Even though there are more than nearly 20 MCDM models available, the derived factor weights based on these models are still subjective^14^. Several bivariate and multivariate statistical models such as frequency ratio^15^, logistic regression^16^, weights of evidence^17^, and certainty factor^18^ also used for generating GESM. The benefit of employing statistical models is that various types of predictor variable can be easily accommodated in the evaluation^13^. The disadvantages of using simple bivariate models are that these could be ad-hoc processes owing to the poor probability distribution that the bivariate models depend on^15^. In the case of parametric multivariate models, the resultant spatial maps become smoother than in MLAs, and provide more elaborate maps of GES^19^.

Various MLAs including random forest^20^, logistic model tree (LMT)^13^, support vector machine(SVM)^21^, naive Bayes tree (NBT)^13^, multivariate adaptive regression spline (MARS)^22^, generalized linear model (GLM)^23^, artificial neural network (ANN)^24^, boosted regression tree (BRT)^22^, mixture discriminant analysis (MDA)^18^, classification and regression trees (CART)^25^, and functional data analysis^14^ are commonly utilized for the creation of GESM. The MLAs exhibit a superior predictive accuracy than statistical models in GESM owing to their advantage in handling huge datasets and potential ability in assessing the intricate relationship between dependent and predictor variables^26^. Performance of individual models can be enhanced using hybrid ensemble methods^27,28^. Hybrid ensemble methods outperform the forecast preciseness of individual MLA^29^. Arabameri et al.^30^ showed that meta-classifiers increase the classification accuracy of the base classifiers in gully erosion susceptibility modelling. It is essential to test a novel base classifier using different meta-classifiers^11^. Chowdhuri et al.^31^ reported high predictive accuracy of hybrid ensemble BRT-bagging (BA) algorithm in comparison with the individual BRT and bagging algorithms. Similar results were displayed by Roy and Saha^32^ in their study in which the authors reported Multilayer perceptron neural network-dagging (DA) ensemble.

In this study, we propose novel hybrid ensemble models for mapping GES based on a case study on the Bastam sedimentary plain of Northern Iran. Apart from individual credal decision trees (CDT) model, we integrated four meta-classifiers including bagging, dagging, rotation forest (RF) and alternating decision tree (ADTree) with a base-classifier, i.e., the CDT for GESM. To our knowledge, no previous study has employed the CDT both as a base classifier in a hybrid ensemble model and as an individual model for predicting the GES. The four hybrid ensemble models, namely CDT-BA, CDT-DA, CDT-RF and CDT-ADTree along with CDT were compared, and the best model is identified. The significance of the gully erosion conditioning factors (GECFs) for mapping GES is evaluated using the random forest model. The predictor variables used in this work for forecasting GES include clay content, bulk density, elevation, distance to road, distance to stream, drainage density, lithology, land use/land cover (LU/LC), normalized difference vegetation index (NDVI), rainfall, terrain rugged index (TRI), slit content, slope degree, and topography wetness index (TWI).

## Results

### Outcome of multi-collinearity test

The values of VIF and tol used for testing the multi-collinearity among GECFs are given in Table 2. The NDVI shows minimum VIF value of 1.099 and TRI has maximum VIF value of 4.184 and, since the tol is the reciprocal of VIF, the NDVI and TRI acquired the maximum (0.910) and minimum tol value (0.239). The VIF and tol values of GECFs from Table 1 indicate that there is no linear dependency among the GECFs and confirms that all the selected fourteen GECFs can be utilized for the generation of GESMs (Table 2).

**Table 1:** Multi-Collinearity analysis of the gully conditioning factors.

Conditioning factors	Collinearity Statistics	
	Tolerance	VIF
Landuse	0.544	1.838
Lithology	0.885	1.130
Elevation	0.304	3.290
TWI	0.817	1.224
Rainfall (mm)	0.260	3.847
Content of silt (%)	0.366	2.736
Slope degree	0.272	3.676
TRI	0.239	4.184
Bulk density	0.455	2.196
Content of clay (%)	0.713	1.403
Distance to road (m)	0.459	2.177
Distance to stream (m)	0.717	1.394
Drainage density (km/km^2^)	0.659	1.518
NDVI	0.910	1.099

**Table 2:** Confusion matrix from the RF model (0 = no gully, 1 = gully).

	0	1
0	190	16
1	11	196

### Relative significance of GECFs

This study employed the random forest algorithm for assessing the significance of GECFs in mapping GES. The confusion matrix created by random forest with gully presence (1) and gully absence (0) information is provided in Table 3. The algorithm generated an OOB error of 6.54%, which infers that the precision of the predicted values is equivalent to 93.46%. From Table 2, it can be observed that among 201 non-gully locations, 190 were identified as non-gully locations and 11 were determined to be gully locations. On the other hand, among 212 gully locations, 196 were predicted as gully locations while 16 were identified as non-gully locations. The outcome of the relative significance of GECFs assessed using the mean decrease in accuracy and mean decrease Gini of the random forest algorithm is provided in Table 3. The GECFs including drainage density (29.10), distance to road (24.72), rainfall (12.86) and NDVI (12.74) exhibited high significance in influencing GE while slope degree (9.48), elevation (9.05), silt content (6.57), bulk density (6.27), TWI (5.79), TRI (5.55) displayed moderate control over the process, but factors such as lithology, clay content, distance to stream and LU/LC showed the least significance in the initiation of GE.

**Table 3:** Relative influence of effective conditioning factors in the random forest model.

Factor	Relative weight	Factor	Relative weight
Drainage density	29.10	Lithology	2.63
Distance to road	24.72	TWI	5.79
Rainfall	12.86	Bulk density	6.27
NDVI	12.74	Slope degree	9.48
Silt content (%)	6.57	Clay content (%)	2.59
Elevation	9.05	LU/LC	0.94
TRI	5.55	Distance to stream	2.51

### Gully erosion susceptibility mapping (GESM)

**Figure 1:**
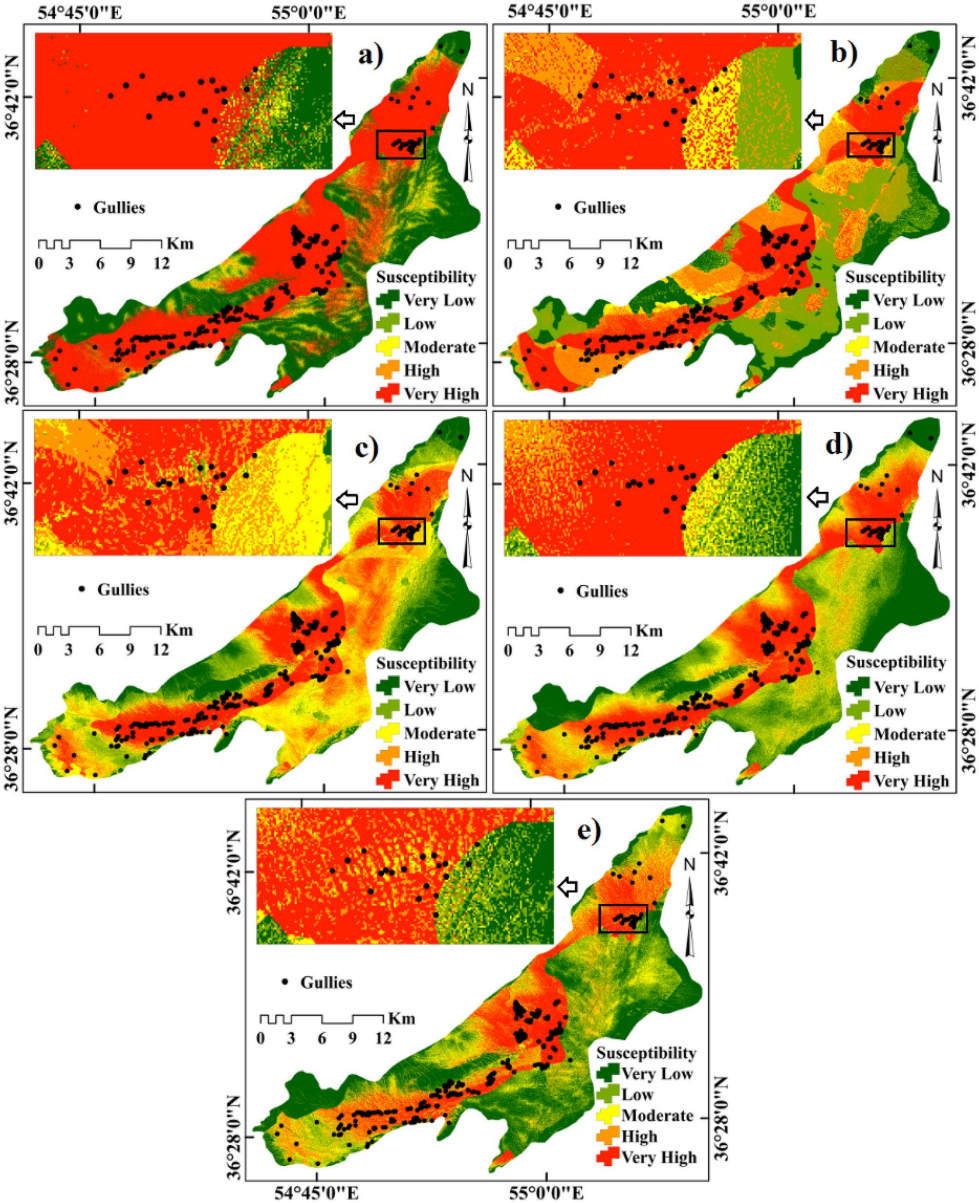
Gully erosion susceptibility mapping using (**a**) credal decision tree (CDT), (**b**) CDT-Dagging, (**c**) CDT-ADTree, (**d**) CDT-Bagging, (**e**) CDT-rotational forest (RF). ArcGIS 10.5 software was used for preparing this map (https://desktop.arcgis.com/en/).

Observations on the presence or absence of gully comprising the values of GECFs were provided as inputs for MLAs in R 3.6.0 to generate the GESMs. The GES index output generated by the CDT, CDT-DA, CDT-ADTree, CDT-BA and CDT-RF models (Fig. 1a–e, respectively) were exported to ArcGIS 10.5 and categorized into very low, low, moderate, high and very high susceptibility classes with the help of natural breaks technique.

### Credal decision tree (CDT)

The GESM produced by CDT shows that 51.16% and 1.67% of pixels come under very high and high GES zone, whereas moderate, low and very low GES zone covers 4.52%, 10.95% and 31.70% of pixels in Bastam sedimentary plain, respectively (Fig. 1a). The total number of pixels present in each GES classes of CDT is provided in Table 4. The number of gully pixels in the very high, high, moderate, low and very low GES zones are279, 4, 3, 2, and 5 whereas the percentage of gully pixels in the same order of susceptibility classes was 95.22%, 1.37%, 1.02%, 0.68% and 1.71%, respectively.

**Table 4:** Quantitative analysis of gully erosion susceptibility maps.

Model	Class	Number of gully pixels	Percentage of class pixels (%)	Percentage of gully pixels (%)
CDT-Dagging	Very low	4	19.19	1.37
	Low	8	22.10	2.73
	Moderate	14	9.10	4.78
	High	42	17.06	14.33
	Very high	225	32.55	76.79
CDT	Very low	5	31.70	1.71
	Low	2	10.95	0.68
	Moderate	3	4.52	1.02
	High	4	1.67	1.37
	Very high	279	51.16	95.22
CDT-ADTree	Very low	3	15.37	1.02
	Low	8	14.74	2.73
	Moderate	10	21.93	3.41
	High	49	21.20	16.72
	Very high	223	26.75	76.11
CDT-Bagging	Very low	2	23.02	0.68
	Low	9	19.59	3.07
	Moderate	14	16.43	4.78
	High	45	15.85	15.36
	Very high	223	25.11	76.11
CDT-RF	Very low	2	28.88	0.68
	Low	11	21.19	3.75
	Moderate	20	15.55	6.83
	High	56	13.64	19.11
	Very high	204	20.74	69.62

### CDT-dagging (DA)

The GESM from CDT-DA model shows about 32.55%, 17.06%, 9.10%, 22.10% and 19.19% of pixels in the study area that falls under very high, high, moderate, low and very low GES class, respectively (Fig. 1b). The percentage of gully pixels present in very high to very low GES classes are 76.79%, 14.33%, 4.78%, 2.73% and 1.37%, respectively (Table 4). The very high and high GES categories comprise 225 and 42 gully pixels whereas the moderate, low and very low GES categories comprised 14, 8, and 4 gully pixels, respectively. The total quantity of pixels in each GES zones of CDT-DA model is shown in Table 4.

### CDT-alternative decision tree (ADTree)

In the case of GESM generated by CDT-ADTree, the percentage of pixels covering very high and high GES categories are 26.75% and 21.20% whereas those of other GES categories including moderate, low and very low classes were 21.93%, 14.74%, and 15.37%, respectively (Fig. 1c). The percentage of gully pixels in very low, low, moderate, high and very high GES regions is 76.11%, 16.72%, 3.41%, 2.37%, and 1.02% whereas the number of gully pixels present in the same order of GES regions was 223, 49, 10, 8 and 3, respectively (Table 4). The information on the number of pixels in each susceptibility class of CDT-ADTree model is given in Table 4.

### CDT-bagging (BA)

The GESM predicted by CDT-BA (Fig. 1d) reveals that percentage of pixels covered by very high, high, moderate, low and very low GES classes are25.11%, 15.85%, 16.43%, 19.59%, and 23.02%, whereas the percentage of gully pixels present in the same order of GES classes are 76.11%, 15.36%, 4.78%, 3.07% and 0.68%, respectively (Table 4). The number of gully pixels existed in the same order of GES classes are 223, 45, 14, 9, and 2, respectively. The number of pixels present in each category of GES generated by CDT-BA is displayed in Table 4.

### CDT-rotational forest (RF)

The GESM generated by CDT-RF shows that 20.74%, 13.64%, 15.55%, 21.19%, and 28.88% of pixels belong to very high, high, moderate, low, and very low GES classes, respectively (Fig. 1e). There are 69.92%, 19.11%, 6.83%, 3.75%, and 0.68% of gully pixels in very high, high, moderate, low and very low GES classes whereas the number of gully pixels in the same order are 204, 56, 20, 11, and 2, respectively (Table 4).

### Outcome of validation measures and model comparison

**Figure 2:**
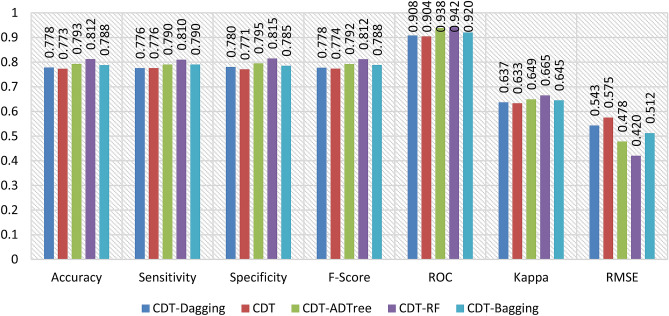
Training performance of the models.

In this study, we assessed the predictive performance of CDT-DA, CDT, CDT-ADTree, CDT-RF, and CDT-BA models with the help of different validation metrics such as accuracy, sensitivity, specificity, F-score, AUROC, Cohen’s Kappa, and RMSE using both calibration (Fig. 2) and testing dataset (Fig. 7).

**Figure 3:**
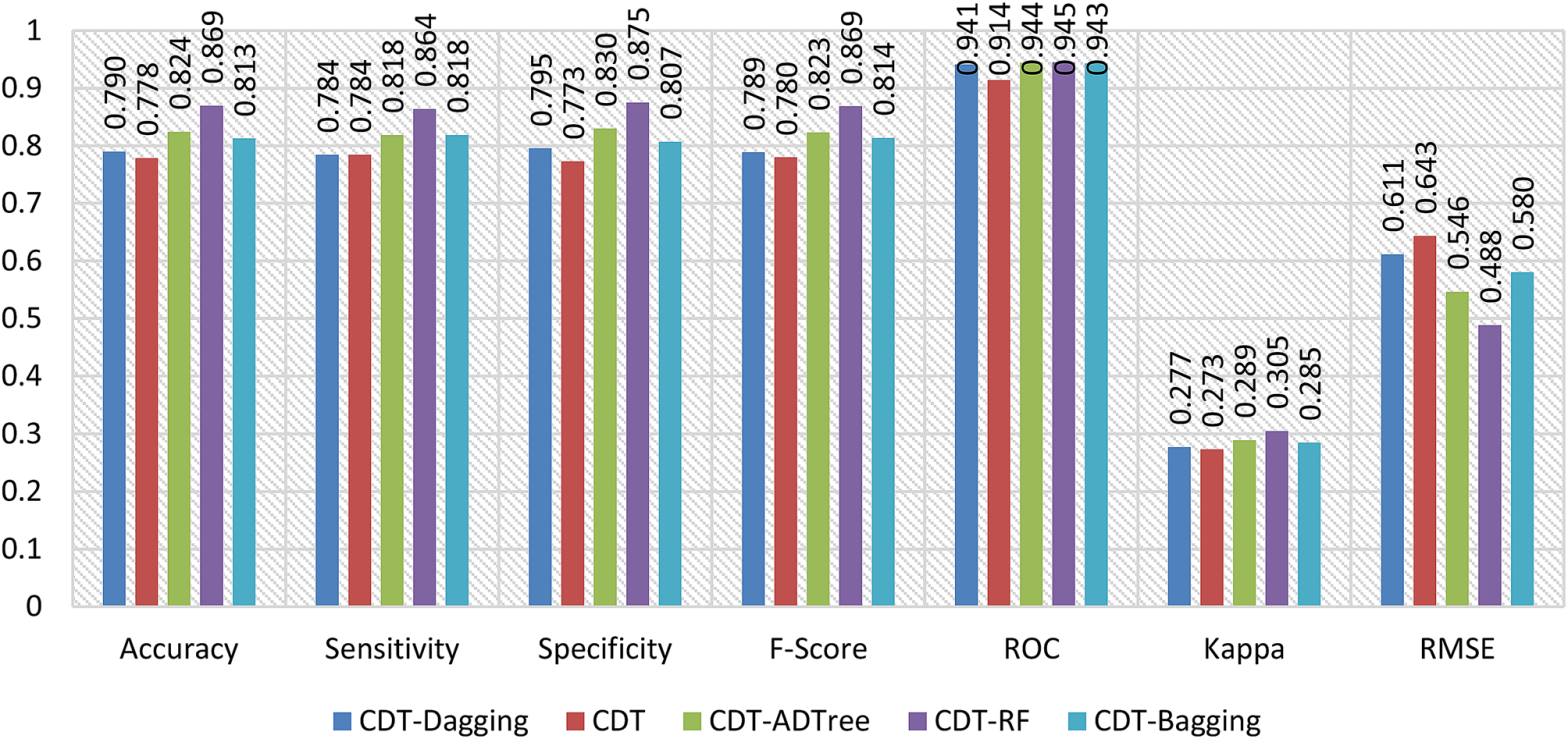
Validation performance of the models.

**Figure 4:**
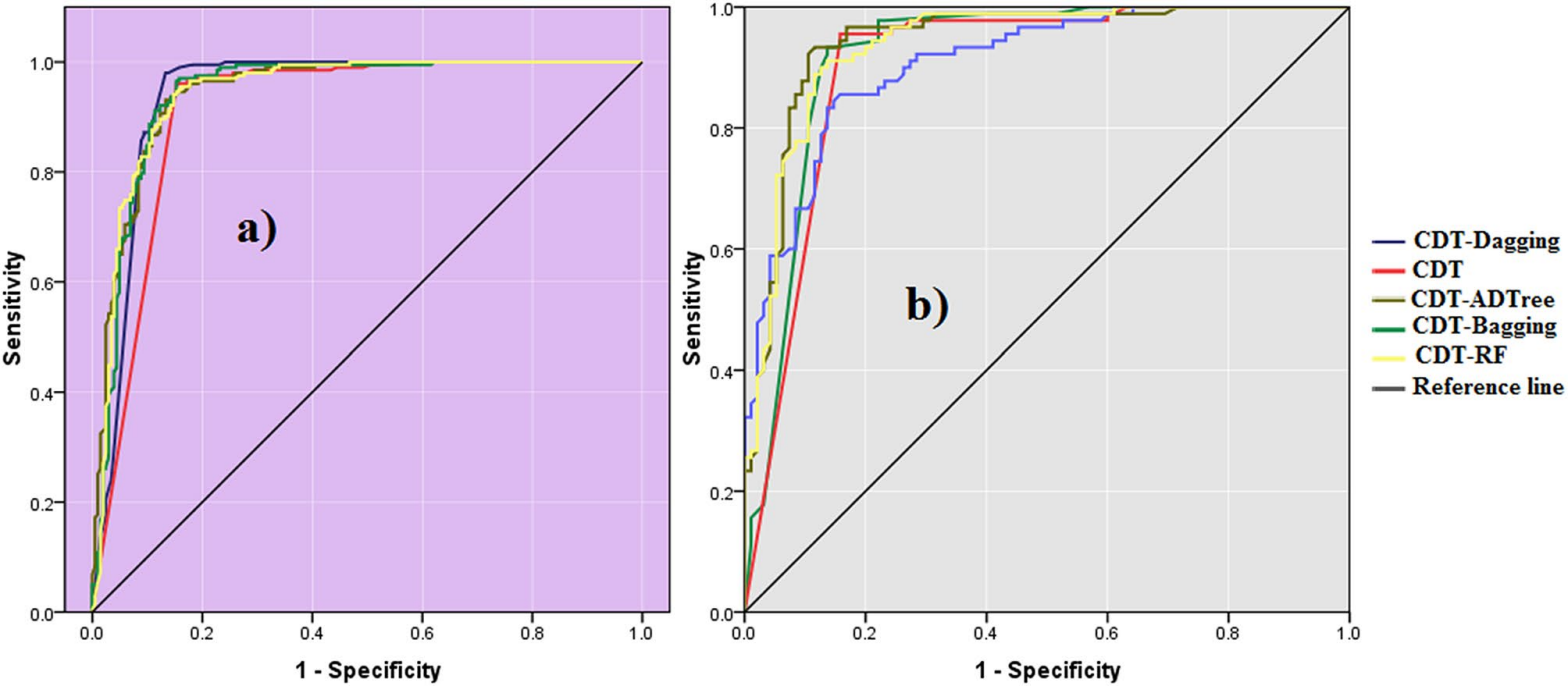
Area under the curve of the models in the training and validation data.

The AUROC curve value of CDT-DA, CDT, CDT-ADTree, CDT-RF, and CDT-BA models using calibration dataset are 0.908, 0.904, 0.938, 0.942, and 0.920 (Figs. 2 and 4a) whereas the values are 0.941, 0.914, 0.944, 0.945, and 0.943 using training dataset, respectively (Figs. 3 and 4b).

Based on calibration dataset, the accuracy of CDT-DA, CDT, CDT-ADTree, CDT-RF, and CDT-BA models are 0.778, 0.773, 0.793, 0.812, and 0.788 (Fig. 2) and using validation dataset the accuracy is 0.790, 0.778, 0.824, 0.869, and 0.813, respectively (Fig. 3). The sensitivity of CDT-DA, CDT, CDT-ADTree, CDT-RF, and CDT-BA models using calibration dataset are 0.776, 0.776, 0.790, 0.810, and 0.790 and specificity is 0.780, 0.771, 0.795, 0.815, and 0.785, respectively (Fig. 2). On the other hand, the sensitivity of CDT-DA, CDT, CDT-ADTree, CDT-RF, and CDT-BA models using testing dataset are 0.784, 0.784, 0.818, 0.864, and 0.818 and specificity is 0.795, 0.773, 0.830, 0.875, and 0.807, respectively (Fig. 3). Using calibration dataset, F-score of CDT-DA, CDT, CDT-ADTree, CDT-RF, and CDT-BA models are 0.778, 0.774, 0.792, 0.812, and 0.788 (Fig. 2) whereas using testing dataset, the F-score values were 0.789, 0.780, 0.823, 0.869, and 0.814, respectively (Fig. 3). The values of Cohen’s Kappa for CDT-DA, CDT, CDT-ADTree, CDT-RF, and CDT-BA models are 0.637, 0.633, 0.649, 0.665, and 0.645 using training dataset (Fig. 2 and with testing dataset, the values are 0.277, 0.273, 0.289, 0.305, and 0.285 (Fig. 3), respectively.

**Figure 5:**
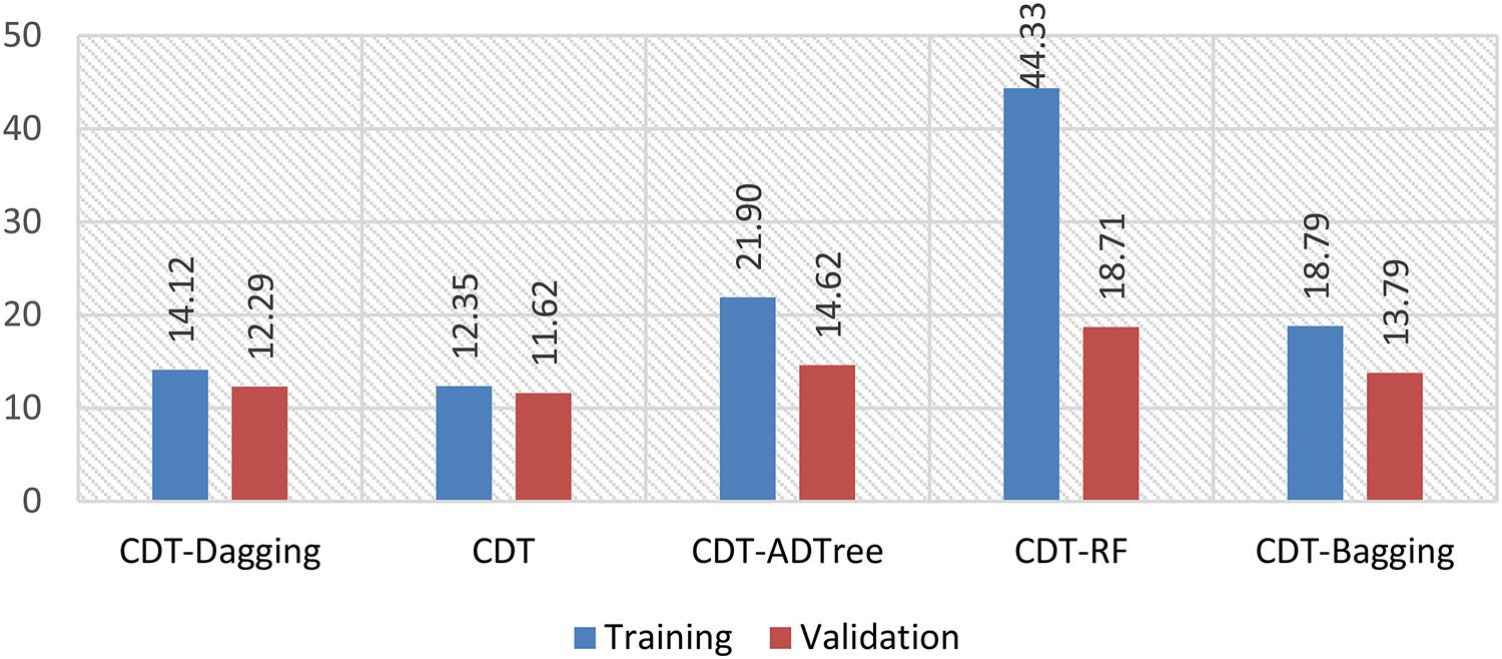
Odd ratio values of the models in the training and validation phases.

While using calibration dataset, the RMSE of CDT-DA, CDT, CDT-ADTree, CDT-RF, and CDT-BA models are 0.543, 0.575, 0.478, 0.420, and 0.512 (Fig. 2) and with testing dataset, the values are 0.611, 0.643, 0.546, 0.488, and 0.580, respectively (Fig. 3). The odd ratio values of the CDT-DA, CDT, CDT-ADTree, CDT-RF, and CDT-BA models in training phase are 14.12, 12.35, 21.90, 44.33, and 18.79 whereas in testing phase the values of odd ratio are 12.29, 11.62, 14.62, 18.71, and 13.79, respectively (Fig. 5). The outcome of validation techniques including accuracy, sensitivity, specificity, F-score, AUROC, Cohen’s Kappa, odd ratio and RMSE displayed the excellent predictive ability of models in mapping GES. Based on the training and testing performance of the models, it is found that CDT-RF was the best model followed by CDT-ADTree, CDT-BA, CDT-DA and CDT models.

**Figure 6:**
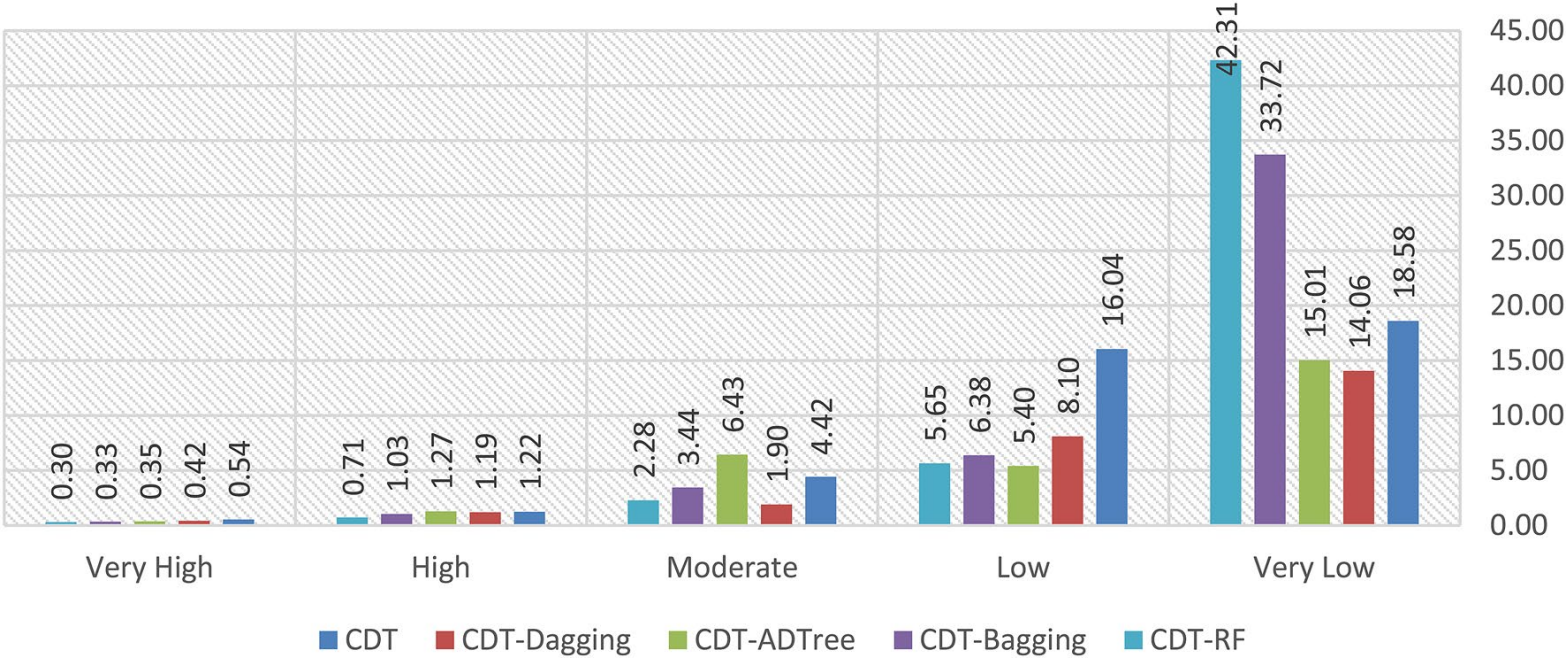
Values of seed cell area index (SCAI) in the susceptibility classes.

The values of SCAI (Fig. 6) generated from GES of CDT-DA, CDT, CDT-ADTree, CDT-RF, and CDT-BA models increased from very high to very low susceptibility. This outcome of SCAI reveals the enhanced predictive performance of the GES models employed in this study.

## Discussion

In recent years, various machine learning^33–36^, Fuzzy^37–41^, deep learning^42–47^, and multiple criteria decision making (MCDM) models^47,48^ along with remote sensing^49–53^ and geographic information system (GIS)^54–56^ have been developed with application in various scientific fields.

Even though the newly developed approaches have advanced from traditional statistical techniques to the MLAs^57–60^, recent studies attempt to formulate novel/hybrid models that could achieve better predictive performance than previously employed approaches. Thus, several studies have successfully enhanced the forecast ability of the MLAs by employing diverse novel ensemble methods. In this study, study we presented a novel hybrid ensemble for GESM in Bastam sedimentary plain of Northern Iran. We employed five MLAs for modelling GES among which four were novel hybrid ensemble models constructed by combining BA, DA, ADTree and RF meta-classifiers with the CDT base classifier and another was an individual CDT. To our knowledge, the hybrid ensembles used in this research to model GES have been not implemented in any other GESM study. Fourteen GECFs including clay content, bulk density, elevation, distance to road, distance to stream, drainage density, lithology, LU/LC, normalized difference vegetation index (NDVI), rainfall, terrain rugged index (TRI), slit content, slope degree and topography wetness index (TWI) were chosen for the modelling of GES. The dependency test among the GECFs was carried out which exposed that there was no correlation, thus making it applicable for processing the outcome.

The importance of GECFs in modelling GES was assessed using the random forest algorithm, which revealed that drainage density, distance to road, rainfall and NDVI were the most influential factors of GES whereas slope degree, elevation, silt content, bulk density, TWI and TRI exhibited moderate control over the GES. Similarly, Pourghasemi et al.^8^ showed that drainage density, distance to stream, soil content and altitude largely influence the initiation of GE. Likewise, Arabameri et al.^61^ determined distance to stream and distance to road to influence the GES most. Capra et al. (2009)^62^ reported that formation of GE is higher when the vegetation cover decreases, and soil wetness increases due to high rainfall. Kariminejad et al.^63^ determined that silt content and slope angle influence GES. Arabameri et al.^11^ showed that topographic factors such as TWI, TRI and elevation has moderate control over the instigation of GE.

The process-response of a river catchment area is highly influenced by several environmental factors, among which drainage is the most vital one, which has a strong positive correlation with gully head cut retreat^11^. The pattern of drainage is also critical in the initiation and further development of gullies. The drainage pattern in a river catchment area is highly affected by nature and structure of the geological formation, soil characteristics, density of vegetation coverage, infiltration rate, and slope degree^22^. Previous studies on gully erosion have shown that initiation and development of gullies are connected to the stream networks and gullying by streams are responsible where favorable conditions are available for their development^20^. The slope instability of an area is causes by initiation of river and the associated toe erosion and fluctuations of groundwater level. Moreover, the degree of surface incision is highly dependent on the pattern of drainage network of an area. The development and pattern of drainage of an area is directly related to the power of degree of surface incision^22^. The road and undercutting construction work gradually increases the strain and stress of the slope which significantly influences slope disturbances and failure^20^. The pattern and rate of surface runoff is mainly determined through road networks, and the concentrated surface runoff flow from one catchment area to another leads to steady increase in watershed size which is ultimately responsible for the process of gullying^20^. The major finding of this research is that CDT-RF (AUSRC = 0.942, AUPRC = 0.945, accuracy = 0.869, specificity = 0.875, sensitivity = 0.864, RMSE = 0.488, F-score = 0.869 and Cohen’s Kappa = 0.305) was determined to be the finest model having superior accuracy than the rest of the hybrid models. The CDT-RF is followed by CDT-ADTree, CDT-BA, CDT-DA and CDT. This clearly shows that RF meta-classifier enhances the predictive performance of individual CDT model. It is also true in the case of other meta-classifiers, namely ADTree, BA and DA, which improves the forecast accuracy of the base classifier. The higher performance of RF can be due to utilization of the feature abstraction method to augment the learning groups for calibrating the base classifiers.

The low predictive accuracy of CDT can be owing to the subset in that the sub-dataset formed is dissimilar from a particular issue field which generates fairly diverse trees^64^. It should also be noted that RF is a powerful MLA that is derived from random forest algorithm. He et al.^65^ also showed that RF increases the predictive ability CDT than any other meta-classifiers such as BA and multiBoostAB (ABM). Nguyen et al.^66^ also determined that different meta-classifiers ABM and radial basis function network (RBFN) increases the forecast ability of CDT. Similarly, both Pham et al.^67^ and Nguyen et al.^68^ demonstrated that meta-classifier helps base classifier CDT in improving the predictive performance in modelling landslide and flash flood vulnerability. From the present study, it is evident that combining meta-classifier such as RF, ADTree, BA and DA with the base-classifier such as CDT would increase its performance in accurately predicting GES. The general advantage of meta-classifiers is that it enhances the predictive accuracy of the MLAs, whereas individual CDF performs well even in noisy datasets. The benefit of utilizing BA is that it is most suitable for classifiers with dipping learning curve and it improves the classification accuracy through the creation of different classifications together. The DA also has the capability in reducing the noise. The reason for lower performance of individual CDT may be attributed to the generation of varying trees, which could be owing to the difference in the sub-dataset constructed for a provided issue domain. The integration of RF with CDT could help the base classifier in decreasing the noise and bias which would eventually result in the higher accuracy of the ensemble. However, there are certain limitations in these models such as use of various predictor variables with diverse values which need to be addressed in future studies.

## Concluding remarks

Identifying precise and robust algorithms for decreasing inaccuracies in GESM and demarcating GES zones is crucial. This research employed four novel hybrid ensemble models (CDT-RF, CDT-ADTree, CDT-BA and CDT-DA) for predicting GES with the aid of fourteen GECFs and 293 gully locations. Various validation measures including SRC, PRC, specificity, sensitivity, Cohen’s Kappa, F-score, accuracy, RMSE and odd ratio were employed for assessing the model outcome using both calibration as well as testing dataset. The outcome of cross-checking revealed that all the employed models had excellent predictive accuracy, among which CDT-RF is identified to be the most robust model. In addition, the outcome of SCAI also suggests the better performance of the models in predicting GES. Our study reveals that meta-classifiers increase the predictive efficacy of base classifiers in modelling GES. The models used in this research can be also applied in other study areas. The GESM generated by CDT-RF model for Bastam sedimentary plain of Northern Iran can therefore be utilized in controlling the occurrence of future gullies and sustainable management of soil resources.

## Methods

### Description of the study area

**Figure 7:**
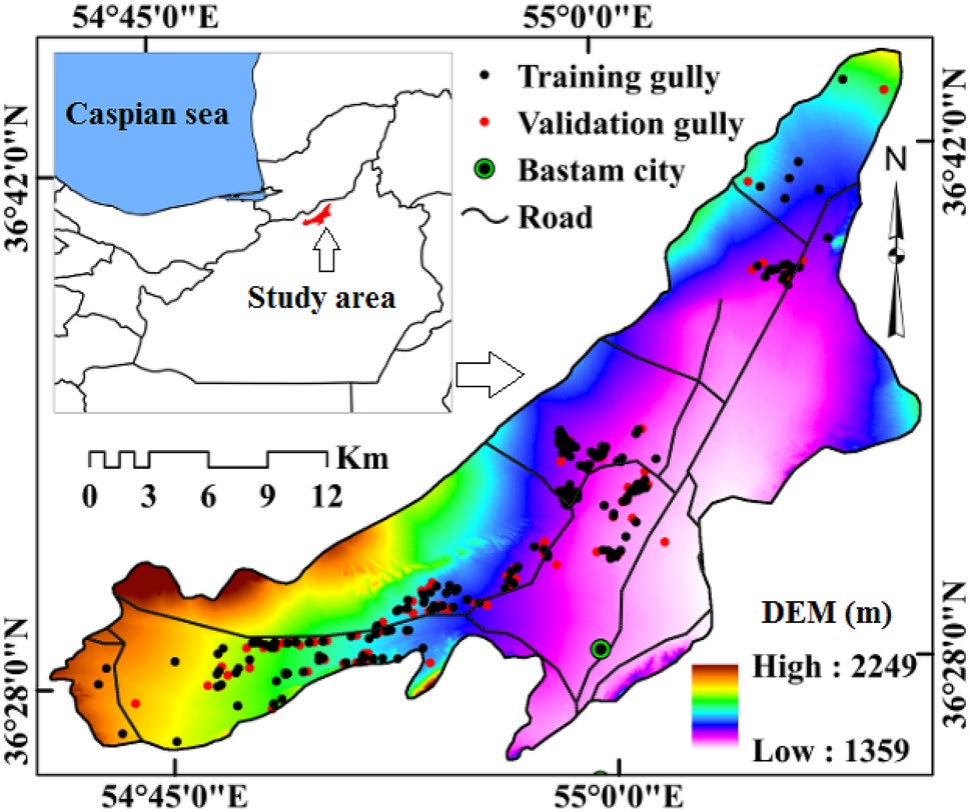
Location of study area in Iran. The map was generated using ArcGIS 10.5 software (https://desktop.arcgis.com/en/).

The Bastam sedimentary plain is one of the most GE prone watersheds located in the Semnan Province of Northern Iran (Fig. 7). It extends between 36° 25′ 53″ N–36° 45′ 43″ N latitudes and 54° 43′ 34″ E–55° 10′ 58″ E longitudes and spreads over an area of about 505.06 km^2^. The average elevation of Bastam sedimentary plain is 1577 m.a.s.l (meters above sea level) where the high and low elevation ranges between 1357 and 2249 m.a.s.l. The high, low and average slope of the study area are 57.96°, 0° and 2.71°, respectively. The annual average precipitation and temperature of this sedimentary plain is 249.5 mm and 14.3 °C, respectively with an arid climate^69^. Different types of land use/land cover (LU/LC) such as rangeland, agriculture, forest, woodland, rock and urban occur in the study area that covers nearly 53%, 44.06%, 2%, 0.49%, 0.66%, 0.185% and 0.72%, respectively of the total area in Bastam sedimentary plain. Rangeland is the dominant vegetation in the study area. The Qal comprising of stream channel, braided channel and flood plain deposits accounts for more than 90% of study area’s lithology^70^ (Table 5). The area is characterized by rock outcrops/entisols, entisols/inceptisols, inceptisols, aridisols and mollisols, covering about 14.77%, 57.11%, 1.61%, 26.33% and 0.14% of the area, respectively^71,72^. Among the several soil types found in the present study area, aridisols cover the maximum portion, constituting the dominant soil type. The evaluation of gullies has indicated that this area is highly susceptible to gully erosion as nearly 10.34% of the study area is affected by ephemeral gully erosion. The low slope area is found to be highly susceptible for gully erosion, with the south-central part more prone to gully erosion as this region is dominated by low slope zone. On the other side, steep slope zone with rocky outcrops in the northern portion of the study area is conquered by a small number of gullies. Morphometric analysis of gullies indicates that the length of gullies ranges from few meters to several hundred meters. The width also varies from few centimeters to several meters and depths can be as much as several meters. The length of the gullies ranges from 364 m (maximum) to 0.95 m (minimum) and depths vary from 6.3 to 0.63 m. Our field survey also reveals that northern part of the study area is dominated by V-shaped cross-section of gullies as this area is characterized by rocky outcrops and steep slope. However, the central and southern parts are dominated by U-shaped gullies, as this area is low slope zone with coverage of more erodible soils and more concentrated runoff and associated erosional activities.

**Table 5:** Lithology of study area.

Unit	Description	Area (km^2^)	Area (%)
DCkh	Yellowish, thin to thick—bedded, fossiliferous argillaceous limestone, dark grey limestone, greenish marl and shale, locally including gypsum	0.37	0.07
Ea.bvs	Andesitic to basaltic volcanosediment	8.94	1.77
Ea.bv	Andesitic and basaltic volcanics	0.03	0.01
K2m, l	Marl, shale and impure limestone	19.67	3.89
Mc	Red conglomerate and sandstone	1.51	0.30
Osh	Greenish—grey siltstone and shale with intercalations of flaggy limestone	18.09	3.58
P	Undifferentiated Permian rocks	454.91	90.07
Qal	Stream channel, braided channel and flood plain deposits	1.55	0.31
TRe2	Thick bedded dolomite	0.37	0.07

## Methodology

**Figure 8:**
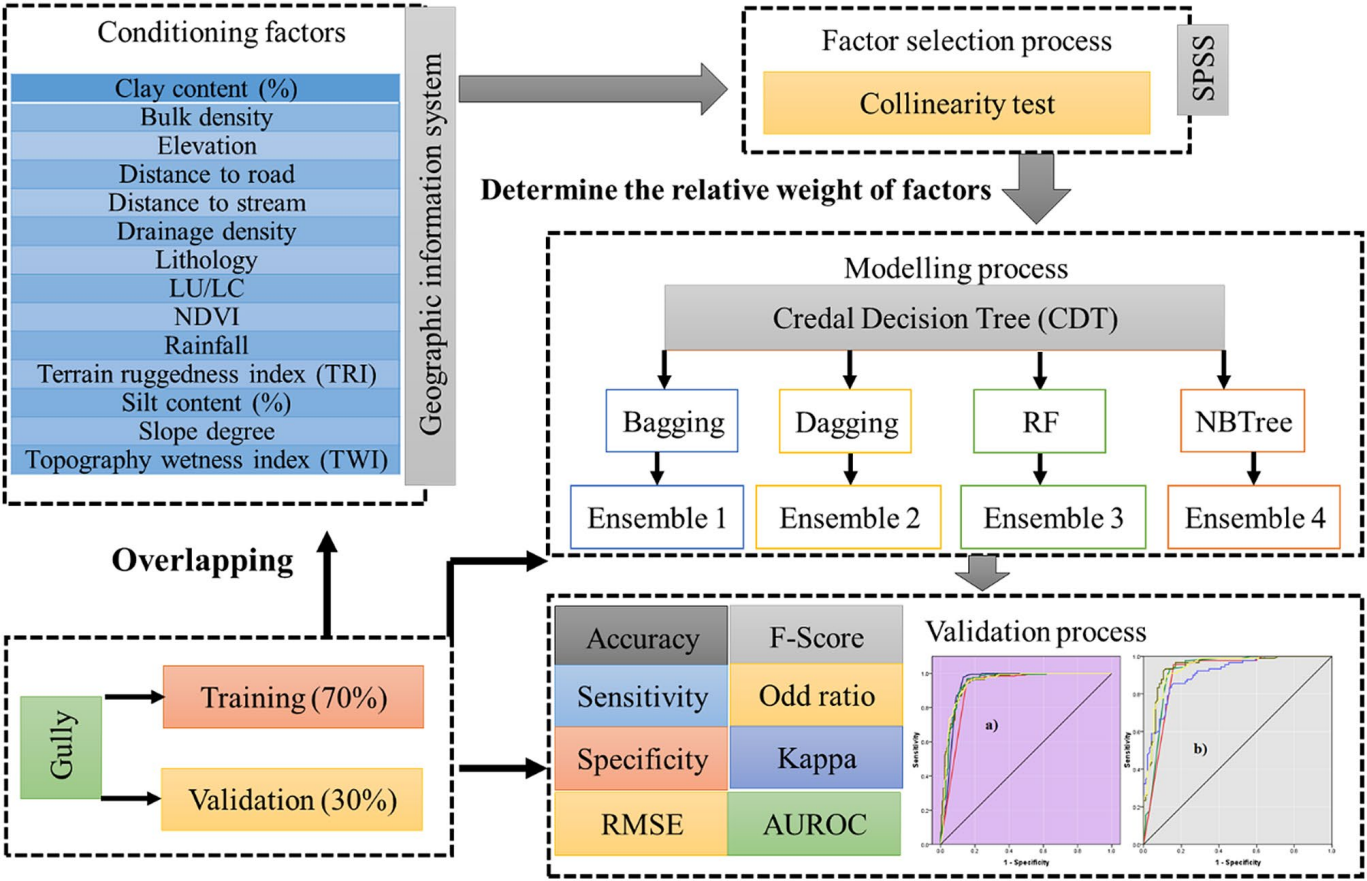
Flowchart of the methodology adopted in the current study.

The mapping of GES with the help of novel ensemble models, including CDT-BA, CDT-DA, CDT-RF and CDT-NBT was executed based on the four following phases (Fig. 8). (1) Initially, the spatial distribution of existing gullies (dependent variable) and GECFs (predictor variables) were prepared for GESM. (2) This was followed by the assessment of multi-collinearity among GECFs. This evaluation is implemented to eliminate noisy GECFs and to confirm that there is no correlation among the predictor variables that could affect the prediction of GE. (3) With the aid of calibration dataset, GESM is generated based on the five models (CDT, CDT-BA, CDT-DA, CDT-RF and CDT-ADTree). The generation of GESMs is followed by the assessment of each independent factor’s influence in predicting the GES using random forest model. 4) Using testing dataset, various validation measures such as the area under receiver operating characteristic curve (AUROC), accuracy, sensitivity, specificity, root mean square error (RMSE), F-score, odd ratio, Cohen Kappa and seed cell area index (SCAI) were applied for cross-checking the predictive ability of the GESM.

### Preparation of gully inventory map

**Figure 9:**
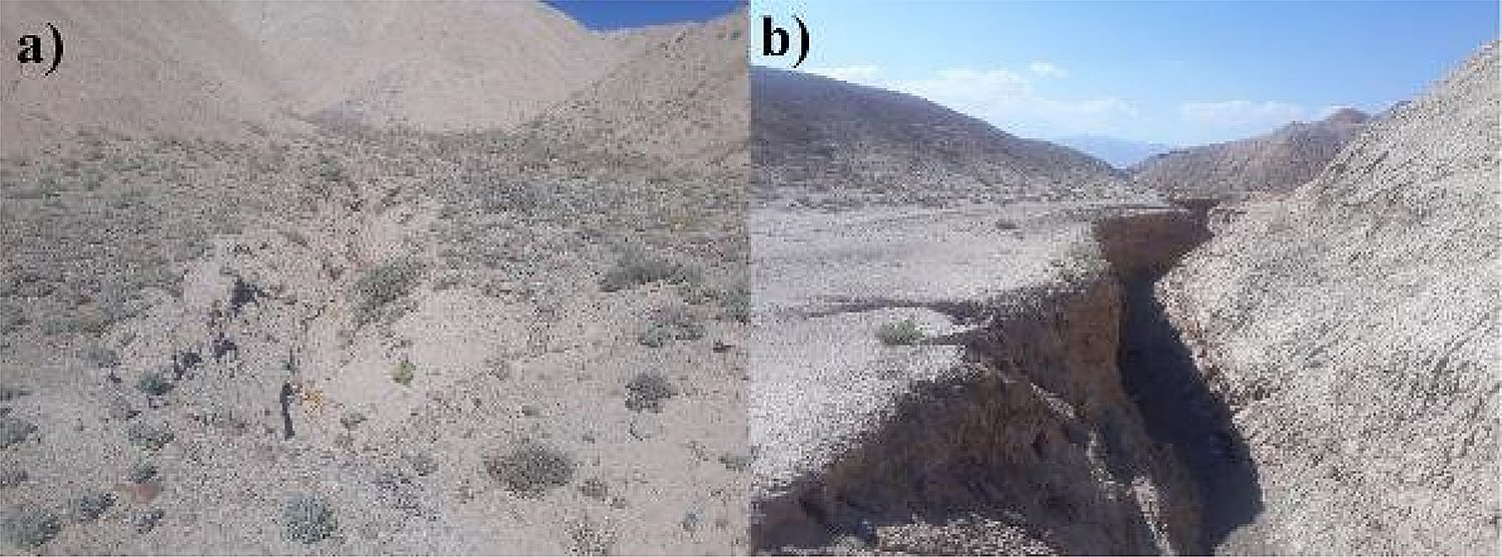
Representative field photographs of the mapped gullies in the study area. (**a**) Lat: 4072920.7; Long 869988.1 (**b**) Lat: 4045271.7; Long 846437.7.

Mapping the extent in the location of gullies in the study area is indispensable for predicting the GES^13^. This is because the susceptibility to most of the natural hazards, including GE is spatially modelled based on the presumption that gullies that occur in future may follow the identical conditions that triggered the existing ones^61^. Thus, understanding the association between the conditioning factors and previously existing gullies are essential^61^. We carried out detailed field investigations using the global positioning system for the preparation of gully inventory map (Fig. 9). A total of 293 gullies were identified in the Bastam sedimentary plain. These were arbitrarily split into 70% (206 gullies) and 30% (87 gullies) for model calibration and testing the predictive ability of the model^13^. In addition, an identical number of non-gully locations were also identified for the processes of model training and validation.

### Preparation of gully erosion conditioning factors

**Figure 10:**
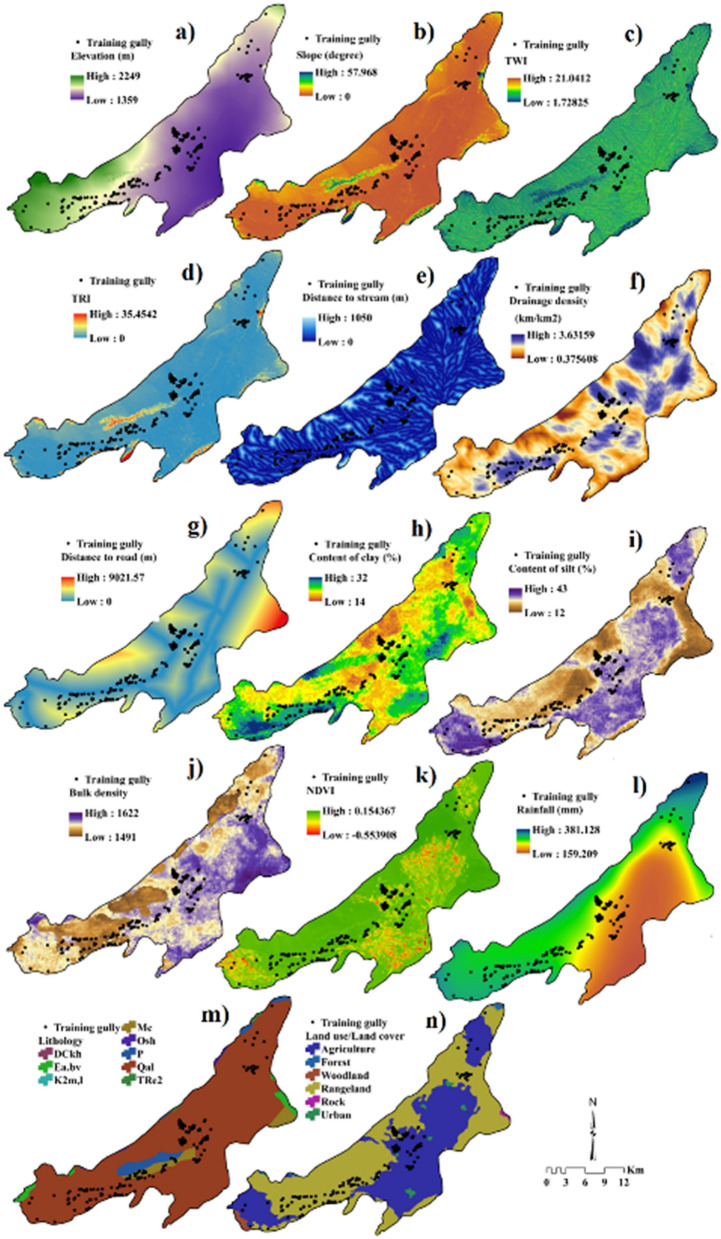
Gully erosion conditioning factors. (**a**) Elevation, (**b**) slope, (**c**) topography wetness index, (**d**) terrain rugged index (TRI), (**e**) distance to stream, (**f**) drainage density, (**g**) distance to road, (**h**) content of clay, (**i**) content of silt, (**j**) bulk density, (**k**) normalized difference vegetation index (NDVI), (**l**) rainfall, (**m**) lithology, (**n**) land use/land cover (LU/LC). The map was generated using ArcGIS 10.5 software (https://desktop.arcgis.com/en/).

GE is an intricate process which is controlled by numerous factors^13,61^ although there are no universally accepted factors that are crucial for GESM^17^. Hence, we carefully selected 14 GECFs from literature review (Fig. 10) namely (a) elevation, (b) slope, (c)TWI, (d)TRI, (e) distance to stream, (f) drainage density, (g) distance to road, (h) content of clay, (i) content of silt, (j) bulk density, (k) NDVI, (l) rainfall, (m) lithology, (n) LU/LC. The GECFs utilized in this research are selected based on the previous investigations, local geo-environmental circumstances and availability of data^11,61,63^. All the 14 GECFs employed in this study were created using ArcGIS 10.5. The primary and secondary topographic factors including elevation, slope degree, TWI and TRI were acquired from ALOS DEM having a spatial resolution of 12.5 m. The stream network and roads were derived from topographical map with a scale of 1:50,000. The 30 years of rainfall data from 9 stations were utilized for the interpolation of rainfall map using Inverse Distance Weighting^63^. Inverse spatial mapping of soil was performed for the areas occupied by gully headcut (GH) morphology. Around 395 soil samples were obtained from the inlets and outlets of GH by digging profile pits ranging between 0 and 2 m in size. While conducting the field investigation, 2 kg of each sample was collected and transported to the lab, where these were air-dried, followed by soil particle size analyses based on the hydrometer technique^71,72^, without eliminating the carbonates, organic matter, and secondary oxides. Secondly, the core approach^73^ was utilized for estimating the bulk density. Following this, the techniques proposed by Walkley and Black (1934)^74^ and Van Bavel^75^ were employed in measuring the organic matter content and stability of the soil. Ultimately, the prepared soil layers were added individually to ArcGIS 10.5 and were processed to the scale of 12.5 m × 12.5 m for additional examination. The foremost soil properties, i.e., bulk density, percentages of silt, and clay content were estimated employing approved petrological techniques and mapped in the GIS.

The lithological units were extracted from maps generated by 1:100,000 (Table 5). The LU/LC of the study area is acquired from Landsat-8 data. Elevation is considered to be a significant factor that influences the occurrence of gullies^13^. It controls the processes of GE owing to its association with various factors such as precipitation, soil texture, run-off, vegetation type and cover^13^. The elevation of Bastam sedimentary plain ranges between 1359 and 2249 m. As slope angle influences runoff and drainage density, it is one of the many important factors that govern gully formation^24^. The slope angle varies from 0 to 57.96%. The TWI is generally applied for assessing the impact of topography on the infusion of water into the saturated zones of runoff generation^24^. TWI is also an effective factor that is essential for GESM owing to its association with soil erosion^11^, and is computed as follows^24^:

1 $$ TWI = \ln \left( {\frac{{D_{s} }}{\tan \mu }} \right) $$

where Ds and μ denote the upslope contributing region and slope incline, respectively. It also aids in assessing the water content present in the soil owing to upstream catchment area and slope^24^. TWI of the Bastam sedimentary plain ranges from 1.728 to 21.04. TRI reflects the terrain morphology and has a considerable effect on surface runoff^24^. TRI values range between 0 and 35.45. Since gully initiation is closely associated with stream networks^61^, the distance to stream plays a major role in gully formation. The maximum and minimum distance to stream was 1050 and 0 m. Drainage density is another important factor to be considered while modelling GES as most of the previous studies have revealed that drainage density is the most influential factor in gully formation^8^. The drainage density of the Bastam sedimentary plain ranges from 0.37 and 3.63 km/km^2^. Building of roads increases the rigidity of gradients, which also leads to gully formation^11^. The minimum and maximum distance to roads are 0 and 9021.57 m. Couper^76^ showed that increase in the content of silt and content of clay would lead to vertical incising of soil, which eventually results in the formation of gullies. The content of clay varies between 32 and 14%, whereas content of silt ranges from 12 to 43%. The increase in the bulk density of soil decreases the potential of plants to reduce the soil erosion. The maximum and minimum bulk density ranges between 1622 and 1491 g cm^−3^. The rainfall is also a significant factor that controls surface flow and erosivity^11^. The high and low rainfall ranges between 381.12 and 159.20 mm. Vegetation cover has an inverse association with soil erosion^8^. In this study, the red band (b4) and infra-red band (b5) from Landsat 8 data were used for the computation of NDVI as follows^8^:

2 $$ {\text{NDVI}} = \, \left( {{\text{b5}} - {\text{b4}}} \right)/\left( {{\text{b5}} + {\text{b4}}} \right) $$

The value of NDVI ranges from -1 to 1, where values < 0.2 indicates non-vegetation and > 0.2 denotes vegetation presence. The NDVI of the study area ranges between 0.15 and − 0.55. The wearing down of bare lithological structures also impacts GE^17^. Table 1 and Fig. 10m provide information of the lithological units existing in the Bastam sedimentary plain. LU/LC is also an important factor considered for GESM^5^. Six types of LU/LC are witnessed in the Bastam sedimentary plain.

### Evaluation of multi-collinearity

It is vital to assess the dependency among the GSCFs before employing these for GESM as the presence of any correlation would impact the consistency and understanding of model outcome^11^. There are numerous techniques including Pearson correlation, variance inflation factors (VIF), ridge regression, the least absolute shrinkage and selection operator (LASSO), conditional index, elastic net, tolerance (tol), and jack-knife tests using which multi-collinearity is evaluated. However, commonly, all multi-collinearity evaluation technique would estimate the dependence between the predictor factors^63^. In this study, we adopted VIF and tol approach for assessing the linear dependency among the GECFs. The expressions of VIF and tol are as follows:

3 $$ tol = 1 - r_{i}^{2} $$

4 $$ VIF = \frac{1}{tol} $$

where $r_{i}^{2}$ is attained by reversing all remaining variables in a multivariate regression^11^. Since there has been no approved values of VIF and tol for denoting the collinearity among predictor variables, commonly established values: tol ≤ 0.1 and VIF ≥ 5 indicates that there is dependency among the independent variables^11^.

### Credal decision tree (CDT)

Abellan and Moral (2003)^77^ introduced CDT for n classification issues through the application of credal sets^78^. It utilizes a unique partitioning condition which was created with the help of uncertainty computation along with inexact possibilities^78^. To circumvent the intricate decision tree (DT) generation while constructing CDT, an innovative idea was developed, which administered to suspend the categorization process from growing the cumulative uncertainty owing to the consequence of DT branching^78^. A modernized approach was developed with the help of the Dempster and Shafe theory, which is utilized for the quantification of overall uncertainty from credal sets^79^. The aforementioned approach is expressed as follows:

5 $$ CU(n) = NC(n) + RC(n) $$

where, *n* denotes a credal set; *CU* signifies the complete uncertainty value; and *NC* and *RC* are functions that refers to the common non-specificity and common randomness, respectively. The creators of CDT obtained series of outcomes and successes compared to *CU* measurement, and furthermore, the computation method of *CU* and its attributes are explained orderly in related sources^79^. The inexact possibility method^78^ was selected to investigate the possibility of interims of discrete variables^79^. Assuming ‘W’ as a variable whose values are denoted with the help of wj, and the identical possibility order p(wj) meets the following expression^79^:

6 $$ p(w_{j} ) \in \left( {\frac{{m_{{w_{j} }} }}{M + h},\frac{{m_{{w_{j} }} + h}}{M + h}} \right) $$

where, m_wj_ refers to the total number of incidence (W = w_j_); M represents the sample size and h denotes the hyperparameter (value: 1 or 2)^79^.

### Bagging (BA)

The BA, also popularly known as bootstrap aggregating, enhances the predictive capabilities of MLAs^80^. Recent studies show that BA has been successfully employed for precise forecasting of susceptibility to various natural hazards^80^. Even a minute variation in the calibration data could create a great difference in the model outcome^80^. BA involves the following stages: (a) arbitrary and independently choosing data from calibration dataset; (b) formation of several classifier models (CMs) with the help of subgroup datasets and (c) model generation through the accumulation of every single CMs^81^. Integrating the rule of base classifiers has been confirmed to have a distinguished impact on BA predicting capability^81^.

Assume C (ai, bi) as a subset of calibration data which is arbitrarily chosen repetitively from a Calibration dataset (ai, bi), where ai represents gully presence and bi refers to gully absence. Multiple CMs are generated based on all subset where Vi(a) represents the created CM. Then finally, every individual classifier (Fi) is combined to form the model outcome (F′). The final prediction of F′ is performed based on the following expression^81^.

7 $$ F^{\prime}(a) = \mathop {\arg }\limits_{b \in B} \max \sum\limits_{i = 1}^{t} {F(V_{i} } (a) = b) $$

### Dagging (DA)

The DA is widely used as an ensemble method that is frequently employed for the creation of meta-classifiers^82^. There are numerous variations between DA and other techniques such as boosting and BA, where boosting flexibly alters the calibration dataset according to distribution while the BA adjust the calibration dataset speculatively and raises bases according to the efficiency of all classifiers as a weight for choosing^82^. In DA, the prediction of a model is carried out based on the top vote^82^. The algorithm utilizes the maximum vote concept for integrating several classifiers in order to enhance the forecast preciseness of the base classifier. DA can be employed in case of base classifiers that are a worst case in timely performance^82^.

### Rotation forest (RF)

The RF is an established integration method which aids weak classifiers in performing better^1,31^. It was introduced by Rodríguez et al.^83^. It is employed in advancing the variation and precision of base classifiers according to the feature transformation^83^. Random forest algorithm serves as the base for the development of RF, still, RF has the improved capability in handling both multi-dimensional and small dataset^83^. The classification possibility of RF algorithm is assessed with the help of the following expressions^83^:

8 $$ v_{\alpha } (a) = \sum\limits_{j = 1}^{l} {f_{m,n} (aS_{j}^{b} )} (j = 1, \ldots ,d) $$

9 $$ a = \arg \max (v_{\alpha } (a))(v \in D) $$

where, a refers to a classification sample; D represents common groups; l indicates the overall quantity of base classifiers and $S_{j}^{b}$ specifies the rotation matrix.

### Alternative decision tree (ADTree)

ADTree was proposed by Freund and Mason (1999) and is by far the highly effective decision tree model which is rooted upon the principle of boosting and is widely applied for modelling purposes^19^. ADT was hardly employed for GESM in previous studies. It provides good accuracy and consistency for categorization and forecast issues^19^. ADTree comprises of two nodes, namely forecast nodes and judgement nodes^19^. The components of a calibration dataset are partitioned into forecast nodes through separation tests, and the equivalent extrapolative values of forecast nodes are acquired. Moreover, through the repetitive estimation, producing and clipping, the ADTree meta-classifier is created that has the affirmative capability to handle intricate and large datasets. The following expression defines the partition testing of forecast node^19^:

10 $$ T(b) = 2(\sqrt {V_{ + } (b)V_{ - } (b)} + \sqrt {V_{ + } ( - b)V_{ - } ( - b))} + V^{\prime} $$

where, V + (b) and V − (b) refers to the complete weight of the calibration data which fulfils the circumstance of c; V′ denotes the overall weight of the dataset which does not fit for the forecast node, and c represents partition testing. The optimal partition testing is attained by determining the least value of T. The appropriate repetitive split test is assessed based on a top to bottom approach in ADTree, and the pruning method applied in this approach is given as follows^19^:

11 $$ T_{pure} = 2(\sqrt {V_{ + } } + \sqrt {V_{ - } } ) + V^{\prime} $$

where, Tpure refers to the lowest threshold of T that is employed for pruning the estimation of few forecast nodes.

### Relative importance assessment of GECFs using random forest

Random forest is a popular non-parametric MLA which comprises a horde of classification and regression trees^61^. Several studies have employed random forest for the evaluation of the significance of predictive variables^84^. RF competently handles vagueness and unknown data and has the exceptional operational ability even with massive and extremely complex datasets^84^. RF comprises two major internal stages. Firstly, it builds several bootstrap samples that are considered to be calibration sets and then constructs classification rules for every tree. In this process, a few datasets that were not employed are leftover; these are known as out-of-bag trials (OOB). OOBs are used to evaluate the inaccuracies in the categorization and to approximate the precision of the prediction^61^.

### Validation measures

Evaluation of the prediction exactness of a model is essential for concluding the technical importance of an investigation^85^. In this study, both training and testing data of GIM is utilized for the cross-checking of the model outcome^1,39^. There are two types of validation metrics, i.e. cut-off-independent and dependent^86^. The computation of validation metrics stated above is executed with the help of contingency table which comprises of four components namely TP (true positive), TN (true negative), FN (false negative), and FP (false positive)^87^. Apart from these measures, SCAI has also been employed in this study to assess the prediction accurateness of the calibrated model.

### Cut-off-independent metrics

The AUROC curve is an extensively utilized metric in various branches of science for accuracy and efficacy evaluation of predictive model outcomes^88,89^. It plots the sensitivity on the Y-axis and 1- specificity on the X-axis^90^. The value of AUROC varies between 0 and 1, where the value equivalent to unity signifies perfect predictive capability^87^. In this research, assessment of success rate curve (SRC) and prediction rate curve (PRC) were carried out using the calibration and testing data of GIM, where the former is employed to estimate the learning ability of the algorithm whereas the latter is applied to determine the forecast capability^90^. The only difference between PRC and SRC is that testing data is replaced with calibration data in PRC^89^.

### Cut-off-dependent metrics

The measures such as accuracy, sensitivity, specificity, F-score, odd ratio and Cohen Kappa belongs to the cut-off dependent approach^89^. The sensitivity refers to the possibility of predicting the gullies precisely as witnessed in actuality, whereas the specificity targets to approximate the likelihood of predicting non-gullies as perceived in actuality^20^. The accuracy represents the efficacy of the model as it reveals the complete success of the forecast model. The F-score is defined as the harmonic average of precision and recall. The values of F-score varies between 0 and 1 where value near 1 represents high precision and recall. Odd ratio estimates the chances that an outcome will appear provided a selective display, related to the chances of the outcome happening in the nonexistence of that display^30^. Cohen’s Kappa tests the robustness of the model and aids the modeller to completely comprehend the actual model outcome^32^. These cut-off-dependent approaches were utilized for assessing both the training as well as the testing performance of the models used in this study. The following expressions are employed for the computation of cut-off-dependent metrics^20^:

12 $$ TPR(sensitivity) = \frac{TP}{{TP + FN}} $$

13 $$ Specificity = \frac{TN}{{TN + FP}} $$

14 $$ accuracy = \frac{(TN + TP)}{{(TN + FP + FN + TP)}} $$

15 $$ F - score = \frac{2TP}{{2TP + FP + FN}} $$

16 $$ odd\mathop {}\limits_{{}} ratio = \frac{TP \times TN}{{FN \times FP}} $$

17 $$ Cohen^{\prime}s\mathop {}\limits_{{}} kappa = \frac{(TP + TN) - [(TP + FN)(TP + FP) + (FN + TN)(FP + TN)]/(T)}{{(T) - \{ [(TP + FN)(TP + FP) + (FN + TN)(FP + TN)]/(T)\} }} $$

### Seed cell area index (SCAI)

Süzen and Doyuran^91^ introduced the SCAI method which is known as the proportion between the total amount of pixels of the particular GES category and the total amount of pixels of prevailing gullies in that particular GES category^86^. Numerous studies have employed SCAI for assessing the performance of the forecast models^20^. The very high value of SCAI for very high susceptibility class and low value of SCAI for low susceptibility class indicates a perfect model and any contrary outcome of this values denotes the poor predictive performance of the model.

### Statistical measures

The RMSE is employed in this study for the validating the model’s calibration as well as testing performance. The RMSE of 0.7 and below indicates better predictive ability while a value greater than 0.7 signifies the poor predictive performance of the model^20,32^. The RMSE is assessed using the following expression:

18 $$ RMSE = \sqrt {1/z\sum\limits_{b = 1}^{z} {(V_{p} - V_{a} )^{2} } } $$

where, Vp refers to the value present in calibration or testing data; Va represents the forecast values produced for the GESMs and z indicates the total number of calibration or testing data.

## References

[1] Sartori M (2019). A linkage between the biophysical and the economic: Assessing the global market impacts of soil erosion. Land Use Policy.

[2] Poesen J (2018). Soil erosion in the Anthropocene: Research needs. Earth Surf. Process. Landforms.

[3] Arabameri A (2020). A methodological comparison of head-cut based gully erosion susceptibility models: Combined use of statistical and artificial intelligence. Geomorphology.

[4] Douglas-Mankin KR (2020). A comprehensive review of ephemeral gully erosion models. CATENA.

[5] Muhs DR (2018). The geochemistry of loess: Asian and North American deposits compared. J. Asian Earth Sci..

[6] Kirkby MJ, Bracken LJ (2009). Gully processes and gully dynamics. Earth Surf. Process. Landforms.

[7] Arabameri A (2018). Spatial modelling of gully erosion using evidential belief function, logistic regression, and a new ensemble of evidential belief function–logistic regression algorithm. L. Degrad. Dev..

[8] Pourghasemi HR, Sadhasivam N, Kariminejad N, Collins AL (2020). Gully erosion spatial modelling: Role of machine learning algorithms in selection of the best controlling factors and modelling process. Geosci. Front..

[9] Poesen, J., Nachtergaele, J., Verstraeten, G. & Valentin, C. Gully erosion and environmental change: Importance and research needs. in *Catena* **50**, 91–133 (Elsevier, 2003).

[10] Rahmati O, Haghizadeh A, Pourghasemi HR, Noormohamadi F (2016). Gully erosion susceptibility mapping: The role of GIS-based bivariate statistical models and their comparison. Nat. Hazards.

[11] Arabameri A, Cerda A, Tiefenbacher JP (2019). Spatial pattern analysis and prediction of gully erosion using novel hybrid model of entropy-weight of evidence. Water.

[12] Zhao J, Vanmaercke M, Chen L, Govers G (2016). Vegetation cover and topography rather than human disturbance control gully density and sediment production on the Chinese Loess Plateau. Geomorphology.

[13] Arabameri A, Chen W, Lombardo L, Blaschke T, Tien Bui D (2020). Hybrid computational intelligence models for improvement gully erosion assessment. Remote Sens..

[14] Arabameri A (2020). Evaluation of recent advanced soft computing techniques for gully erosion susceptibility mapping: A comparative study. Sensors.

[15] Meliho M, Khattabi A, Mhammdi N (2018). A GIS-based approach for gully erosion susceptibility modelling using bivariate statistics methods in the Ourika watershed Morocco. Environ. Earth Sci..

[16] Conoscenti C (2014). Gully erosion susceptibility assessment by means of GIS-based logistic regression: A case of Sicily (Italy). Geomorphology.

[17] Dube F (2014). Potential of weight of evidence modelling for gully erosion hazard assessment in Mbire District, Zimbabwe. Phys. Chem. Earth.

[18] Hosseinalizadeh M (2019). How can statistical and artificial intelligence approaches predict piping erosion susceptibility?. Sci. Total Environ..

[19] Arabameri A (2020). Comparison of machine learning models for gully erosion susceptibility mapping. Geosci. Front..

[20] Saha S, Roy J, Arabameri A, Blaschke T, Tien Bui D (2020). Machine learning-based gully erosion susceptibility mapping: A case study of Eastern India. Sensors.

[21] Amiri M, Pourghasemi HR, Ghanbarian GA, Afzali SF (2019). Assessment of the importance of gully erosion effective factors using Boruta algorithm and its spatial modeling and mapping using three machine learning algorithms. Geoderma.

[22] Arabameri A, Pradhan B, Pourghasemi HR, Rezaei K, Kerle N (2018). Spatial modelling of gully erosion using GIS and R programing: A comparison among three data mining algorithms. Appl. Sci..

[23] Gayen, A. & Pourghasemi, H. R. Spatial Modeling of Gully Erosion: A New Ensemble of CART and GLM Data-Mining Algorithms. in *Spatial Modeling in GIS and R for Earth and Environmental Sciences* 653–669 (Elsevier, 2019). doi:10.1016/b978-0-12-815226-3.00030-2

[24] Garosi Y (2018). Comparison of differences in resolution and sources of controlling factors for gully erosion susceptibility mapping. Geoderma.

[25] Gutiérrez, Á. G., Schnabel, S. & Lavado Contador, J. F. Using and comparing two nonparametric methods (CART and MARS) to model the potential distribution of gullies. *Ecol. Modell.* **220**, 3630–3637 (2009).

[26] Arabameri A, Pradhan B, Lombardo L (2019). Comparative assessment using boosted regression trees, binary logistic regression, frequency ratio and numerical risk factor for gully erosion susceptibility modelling. CATENA.

[27] Cao B, Dong W, Lv Z, Gu Y, Singh S, Kumar P (2020). Hybrid microgrid many-objective sizing optimization with fuzzy decision. IEEE Trans. Fuzzy Syst..

[28] Liu S, Yu W, Chan FTS, Niu B (2020). A variable weight-based hybrid approach for multi-attribute group decision making under interval-valued intuitionistic fuzzy sets. Int. J. Intell. Syst..

[29] Peng S, Zhang Z, Liu E, Liu W, Qiao W (2020). A new hybrid algorithm model for prediction of internal corrosion rate of multiphase pipeline. J. Nat. Gas Sci. Eng..

[30] Arabameri, A. *et al.* Gully head-cut distribution modeling using machine learning methods-a case study of N.W. Iran. *Water (Switzerland)* **12**, 16 (2020).

[31] Chowdhuri I (2020). Implementation of artificial intelligence based ensemble models for gully erosion susceptibility assessment. Remote Sens..

[32] Roy J, Saha S (2020). Integration of artificial intelligence with meta classifiers for the gully erosion susceptibility assessment in Hinglo river basin Eastern India. Adv. Sp. Res..

[33] Fu X, Yang Y (2020). Modeling and analysis of cascading node-link failures in multi-sink wireless sensor networks. Reliab. Eng. Syst. Saf..

[34] Qu S, Han Y, Wu Z, Raza H (2020). Consensus modeling with asymmetric cost based on data-driven robust optimization. Group Decis. Negot..

[35] Tsai Y-H, Wang J, Chien W-T, Wei C-Y, Wang X, Hsieh S-H (2019). A BIM-based approach for predicting corrosion under insulation. Autom. Constr..

[36] Wang S, Zhang K, van Beek LPH, Tian X, Bogaard TA (2019). Physically-based landslide prediction over a large region: Scaling low-resolution hydrological model results for high-resolution slope stability assessment. Environ. Modell. Softw..

[37] Cao B, Zhao J, Lv Z, Gu Y, Yang P, Halgamuge S (2020). Multiobjective evolution of fuzzy rough neural network via distributed parallelism for stock prediction. IEEE Trans. Fuzzy Syst..

[38] Shi K, Wang J, Tang Y, Zhong S (2020). Reliable asynchronous sampled-data filtering of T-S fuzzy uncertain delayed neural networks with stochastic switched topologies. Fuzzy Sets Syst..

[39] Shi, K., wang, J., Zhong, S., Tang, Y. & Cheng, J. Non-fragile memory filtering of T-S fuzzy delayed neural networks based on switched fuzzy sampled-data control. *Fuzzy Sets Syst.*10.1016/j.fss.2019.09.00 (2019).

[40] Wu T, Cao J, Xiong L, Zhang H (2019). New stabilization results for semi-markov chaotic systems with fuzzy sampled-data control. Complexity.

[41] Bui DT, Moayedi H, Gör M, Jaafari A, Foong LK (2019). Predicting slope stability failure through machine learning paradigms. ISPRS Int. J. Geo-Inf..

[42] Xu M, Li T, Wang Z, Deng X, Yang R, Guan Z (2018). Reducing complexity of HEVC: A deep learning approach. IEEE Trans. Image Process..

[43] Chen H, Chen A, Xu L, Xie H, Qiao H, Lin Q, Cai K (2020). A deep learning CNN architecture applied in smart near-infrared analysis of water pollution for agricultural irrigation resources. Agric. Water Manag..

[44] Qian J, Feng S, Tao T, Hu Y, Li Y, Chen Q, Zuo C (2020). Deep-learning-enabled geometric constraints and phase unwrapping for single-shot absolute 3D shape measurement. APL Photon..

[45] Li, T., Xu, M., Zhu, C., Yang, R., Wang, Z. & Guan, Z. A deep learning approach for multi-frame in-loop filter of HEVC. *IEEE Trans. Image Process.* 1–1 (2019). doi:10.1109/tip.2019.2921877. 10.1109/TIP.2019.2921877 31217108

[46] Qiu, T. *et al.* Deep Learning: A rapid and efficient route to automatic meta-surface design. *Adv. Sci.* 1900128 (2019). doi:10.1002/advs.20190012 10.1002/advs.201900128 PMC6662056 31380164

[47] Liu S, Chan FTS, Ran W (2016). Decision making for the selection of cloud vendor: An improved approach under group decision-making with integrated weights and objective/subjective attributes. Expert Syst. Appl..

[48] Wu, C., Wu, P., Wang, J., Jiang, R., Chen, M. & Wang, X. Critical review of data-driven decision-making in bridge operation and maintenance. Struct. Infrastruct. Eng. 1–24 (2020). doi:10.1080/15732479.2020.1833946

[49] Han C, Zhang B, Chen H, Wei Z, Liu Y (2019). Spatially distributed crop model based on remote sensing. Agric. Water Manag..

[50] Zuo C, Chen Q, Tian L, Waller L, Asundi A (2015). Transport of intensity phase retrieval and computational imaging for partially coherent fields: The phase space perspective. Opt. Lasers Eng..

[51] Yan J, Pu W, Zhou S, Liu H, Bao Z (2019). Collaborative detection and power allocation framework for target tracking in multiple radar system. Inf. Fusion.

[52] Zuo C, Chen Q, Gu G, Feng S, Feng F, Li R, Shen G (2013). High-speed three-dimensional shape measurement for dynamic scenes using bi-frequency tripolar pulse-width-modulation fringe projection. Opt. Lasers Eng..

[53] Zhu J, Wu P, Chen M, Kim MJ, Wang X, Fang T (2020). Automatically processing IFC clipping representation for BIM and GIS integration at the process level. Appl. Sci..

[54] Zhu J, Wang X, Wang P, Wu Z, Kim MJ (2019). Integration of BIM and GIS: Geometry from IFC to shapefile using open-source technology. Autom. Constr..

[55] Zhu J, Wang X, Chen M, Wu P, Kim MJ (2019). Integration of BIM and GIS: IFC geometry transformation to shapefile using enhanced open-source approach. Autom. Constr..

[56] Tian, P., Lu, H., Feng, W., Guan, Y. & Xue, Y. Large decrease in streamflow and sediment load of Qinghai–Tibetan Plateau driven by future climate change: A case study in Lhasa River Basin. *CATENA*, 104340 (2019). doi:10.1016/j.catena.2019.104340.

[57] Cao B, Wang X, Zhang W, Song H, Lv Z (2020). A many-objective optimization model of industrial internet of things based on private Blockchain. IEEE Netw..

[58] Feng, W., Lu, H., Yao, T. & Yu, Q. Drought characteristics and its elevation dependence in the Qinghai–Tibet plateau during the last half-century. *Sci. Rep.* **10**(1). doi:10.1038/s41598-020-71295-1 (2020) 10.1038/s41598-020-71295-1 PMC7459105 32868800

[59] Chao L, Zhang K, Li Z, Zhu Y, Wang J, Yu Z (2018). Geographically weighted regression based methods for merging satellite and gauge precipitation. J. Hydrol..

[60] Zhang K (2019). Ground observation-based analysis of soil moisture spatiotemporal variability across a humid to semi-humid transitional zone in China. J. Hydrol..

[61] Arabameri A, Pradhan B, Rezaei K (2019). Gully erosion zonation mapping using integrated geographically weighted regression with certainty factor and random forest models in GIS. J. Environ. Manage..

[62] Capra A, Porto P, Scicolone B (2009). Relationships between rainfall characteristics and ephemeral gully erosion in a cultivated catchment in Sicily (Italy). Soil Tillage Res..

[63] Kariminejad N (2019). Evaluation of factors affecting gully headcut location using summary statistics and the maximum entropy model: Golestan Province NE Iran. Sci. Total Environ..

[64] Abellán J, Masegosa AR (2010). An ensemble method using credal decision trees. Eur. J. Oper. Res..

[65] He Q (2019). Novel entropy and rotation forest-based credal decision tree classifier for landslide susceptibility modeling. Entropy.

[66] Nguyen V-T (2019). GIS based novel hybrid computational intelligence models for mapping landslide susceptibility: A case study at Da Lat City Vietnam. Sustainability.

[67] Pham, B. T. *et al.* GIS based hybrid computational approaches for flash flood susceptibility assessment. *Water (Switzerland)* **12**, 683 (2020).

[68] Nguyen PT (2020). Improvement of credal decision trees using ensemble frameworks for groundwater potential modeling. Sustainability.

[69] I.R. of Iran Meteorological Organization (IRMIO). (2012). Available at: http://www.mazandaranmet.ir. (Accessed: 11th May 2020)

[70] *Geology Survey of Iran (GSI)*. (1992).

[71] IUSS Working Group WRB. *World Reference Base for Soil Resources*. *World Soil Resources Report* (2014).

[72] Beretta AN (2014). Soil texture analyses using a hydrometer: Modification of the Bouyoucos method. Cienc. e Investig. Agrar..

[73] Bernatek-Jakiel A, Wrońska-Wałach D (2018). Impact of piping on gully development in mid-altitude mountains under a temperate climate: A dendrogeomorphological approach. CATENA.

[74] Walkey A, Black IA (1930). An examination of the Degtjareff method for determining soil organic matter, and a proposed modification of the chromic acid titration method. Soil Sci..

[75] van Bavel CHM (1950). Mean weight-diameter of soil aggregates as a statistical index of aggregation. Soil Sci. Soc. Am. J..

[76] Couper P (2003). Effects of silt-clay content on the susceptibility of river banks to subaerial erosion. Geomorphology.

[77] Abellán J, Moral S (2003). Building classification trees using the total uncertainty criterion. Int. J. Intell. Syst..

[78] Mantas, C. J. & Abellán, J. Credal-C4.5: decision tree based on imprecise probabilities to classify noisy data. *Expert Syst. Appl.* **41**, 4625–4637 (2014).

[79] Abellan, J. & Moral, S. A non-specificity measure for convex sets of probability distributions. *Int. J. Uncert. Fuzziness Knowl. Based Syst.* **8**, 357–367 (2000).

[80] Luo X (2019). Coupling logistic model tree and random subspace to predict the landslide susceptibility areas with considering the uncertainty of environmental features. Sci. Rep..

[81] Arabameri A (2020). Flash flood susceptibility modelling using functional tree and hybrid ensemble techniques. J. Hydrol..

[82] Bauer E, Kohavi R (1999). Empirical comparison of voting classification algorithms: bagging, boosting, and variants. Mach. Learn..

[83] Rodríguez JJ, Kuncheva LI, Alonso CJ (2006). Rotation forest: A New classifier ensemble method. IEEE Trans. Pattern Anal. Mach. Intell..

[84] Du P, Samat A, Waske B, Liu S, Li Z (2015). Random Forest and Rotation Forest for fully polarized SAR image classification using polarimetric and spatial features. ISPRS J. Photogramm. Remote Sens..

[85] Nguyen H, Mehrabi M, Kalantar B, Moayedi H, Abdullahi MM (2019). Potential of hybrid evolutionary approaches for assessment of geo-hazard landslide susceptibility mapping. Geomat. Nat. Hazards Risk..

[86] Wang H, Moayedi H, Kok Foong L (2020). Genetic algorithm hybridized with multilayer perceptron to have an economical slope stability design. Eng. Comput..

[87] Xi W, Li G, Moayedi H, Nguyen H (2019). A particle-based optimization of artificial neural network for earthquake-induced landslide assessment in Ludian county China. Geomat. Nat. Hazards Risk..

[88] Rahmati O (2019). Land subsidence modelling using tree-based machine learning algorithms. Sci. Total Environ..

[89] Moayedi H, Khari M, Bahiraei M, Kok Foong L, Bui DT (2020). Spatial assessment of landslide risk using two novel integrations of neuro-fuzzy system and metaheuristic approaches, Ardabil Province. Iran. Geomatics. Nat. Hazards Risk.

[90] Bui DT, Moayedi H, Kalantar B, Osouli A, Pradhan B, Nguyen H, Rashid ASA (2019). A novel swarm intelligence—Harris Hawks optimization for spatial assessment of landslide susceptibility. Sensors.

[91] Süzen ML, Doyuran V (2004). A comparison of the GIS based landslide susceptibility assessment methods: Multivariate versus bivariate. Environ. Geol..

